# On the unethicality of disablism: Excluding intellectually impaired individuals from participating in research can be unethical

**DOI:** 10.4102/ajod.v1i1.23

**Published:** 2012-09-04

**Authors:** Charlotte Capri, Ockert Coetzee

**Affiliations:** 1 Intellectual Disability Services, Lentegeur Psychiatric Hospital, Western Cape, South Africa; 2 Department of Psychology, University of Stellenbosch, South Africa; 3 Department of Psychology, Alexandra Hospital, Western Cape, South Africa; 4 Department of Psychiatry and Mental Health,University of Cape Town, South Africa

## Introduction

As coconstructors of studies that may affect them directly, adults living with intellectual impairment need not be excluded as coresearchers. Assuming that these adults do not have capacity to consent as participants in research due to impaired cognitive functioning presumes incapacity (Dye, Hendy, Hare, & Burton 2004). Exclusion on the basis of impairment could be seen as discriminating and a contravention of a non-derogable human right (*Constitution of the Republic of South Africa* [*RSA*], *No. 108 of 1996*). This could also be construed as unethical since such omissions may hinder rather than enable developments to improve health and services for intellectually impaired persons. As does any South African, intellectually impaired citizens have the right to benefit from scientific progress, and even more so if they can contribute as experts to such progress (London, Kagee, Moodley, & Swartz 2011). By virtue of their expertise on disability matters, their voice may stand in public and scientific service.

In following London and colleagues (2011), a human rights perspective could provide a useful view on the unethicality of either excluding or coercing intellectuality impaired individuals as research participants. All South Africans have a right to equality,^1^ freedom of expression,^2^ health care services,^3^ and human dignity.^4^ Yet it remains the task of the researcher to hold for participants the tension between being included voluntarily and feeling coerced – the right not to be discriminated against whilst upholding that of psychological integrity (RSA 1996). An appreciation of intellectually impaired individuals’ understanding of dignity, (self) respect, and ‘nonhumiliation’ might also contribute to better practice in the process of obtaining consent for participation (Nussbaum 2010: 79).

Opinions of quality of care, resources, and services are very rarely obtained from intellectually impaired individuals themselves; when viewed as incompetent to pass judgement on their own experiences, we may be contravening aforementioned human rights (RSA 1996; Kittay 2009; Tronto 2011). If ‘the preferencing of the interests of vulnerable people and groups in ways that enable them to change the conditions of their vulnerability … is … [p]aramount to a human rights perspective’, we can neither learn how intellectually impaired individuals experience the care quality, resources, and services they receive if not allowed to tell us; nor help raise these expert voices (London *et al*. 2011:3). We see that Kittay (2009) finds it morally abusive when policies impacting intellectually impaired individuals are ‘formulated on the basis of the denial of the moral personhood of individuals who do not have a place at the table where their fates may be decided’ (2009:620).

Individuals may be intellectually or physically impaired, but it is their political and social environments that do the disabling. Swartz (2010) explains that in the social model of disability, the impairment alone ‘is not sufficient for disablement to occur. What disables people – what *makes* people disabled – is how society responds to the impairments’ [*my italics for emphasis*] (Swartz 2010:27-28; see also Walmsley 2001). Not being allowed to ask the opinion of intellectually impaired individuals on matters that affect them directly can be construed as disabling, and may amount to disablist practice. If located in emancipatory research, disability studies must explore ways in which individuals living with intellectual impairment can, as co-researchers, have some measure of control over studies that affect them directly (Barton 2006; Walmsley & Johnson 2003; Walmsley 2001).

Although the fundamental ethical principle of anonymising data might serve to protect the ‘welfare and dignity’ of participants (Marzano 2007:418), not documenting their perception of dignifying experiences could silence a possible lack of these and constitute a disavowal of living and working with intellectual disability – an unacknowledgement that raises further ethical concerns (Nussbaum 2010). How can lived experiences on the continuum of intellectual disability become known and knowable if the bodies these experiences are lived in cannot be named? What if unilaterally deciding to protect participant identities is not in their best interest… *too ashamed to be named*? Shouldn’t anonymity be negotiated with participants as a power issue, something intersubjective work enables (Swartz, Van der Merwe, Buckland, & McDougall 2011)? Assuming that, because of weaker cognitive functioning, intellectually disabled adults lack the capacity to agree or decline to participate in research negates the right they have to inclusion and acknowledgement, and the right to claim the time and thinking of the enabled researcher (Kittay 2009; Sinason 2010).

### Benefits of participation

Disability researchers emphasise the importance of moving studies about impaired persons from a third-person reporting style that continues to disable intellectually impaired voices as subaltern, toward counter-hegemonic discursive texts where the experience and expert voice of impaired individuals are at the core (Dye *et al*. 2004; French & Swain 1997; Swartz *et al*. 2011; Swartz 2010; Walmsley 2004a, 2001). By continuing to take a ‘speaking for’ position rather than one of ‘speaking with’, well-meaning enabled researchers may unwittingly contribute to *scientific silencing* – further incapacitating already subdued voices. By co-creating counter-hegemonic disability texts, intellectually impaired individuals could ensure that disability studies are not dominated by enabled researchers and their agendas, or by issues that are *only* important to professionals (French & Swain 1997; Inglis & Cook 2011; Swartz *et al*. 2011).

In helping to locate novel aspects within disability studies, intellectually impaired coresearchers could assist in preventing inclusive disability research from becoming marginalised (Gilbert 2004; Walmsley 2001). Inclusive research could add depth and strength to data collection; involve participants in effecting political and social processes of change; acknowledge and credit participant opinions, ideas, and insights; and contribute to facilitating participant confidence and self-esteem (Barton 2006; Dye *et al*. 2004; Gilbert 2004; Inglis & Cook 2011; Stone & Priestly 1996). Excluding intellectually impaired individuals from research projects might deny them indirect benefits of pride in having their contributions credited; a sense of achievement and worth gained as coresearchers; intellectual stimulation; additional attention from various professionals; and gaining awareness of their capabilities (Inglis & Cook 2011; Sinason 2010).

### Obstacles to participation

From the outset, the burden of the consent process must be formalised in a research proposal – the onus to obtain consent is on the researcher, not on the participant to provide it. Obtaining participant consent from intellectually impaired individuals presents particular ethical challenges. A significant tension exists between ensuring that participants understand the nature and implications of their research involvement, while avoiding any form of coercion.

Iacano and Murray (2003:49) note that there is ‘a need to protect vulnerable participant groups’, but that there also need to be ways of ensuring that ‘demands placed on researchers are not so restrictive as to preclude valuable research’ (see also Marzano 2007). London and colleagues (2011) highlight the dilemma of restrictive ethical approval processes on research in their example of how various regulatory frameworks specify the nature of information that must be included and understood in a consent form (see also Gilbert 2004). Although understandable from the perspective of political and human rights redress, ‘South African regulatory requirements [Department of Health 2004] specify 27 elements that must be included in a consent document … [i]n the USA, federal regulations require a minimum of eight items. Given these disparate criteria, it is … difficult to establish an acceptable minimum standard of understanding’ (London *et al*. 2011:3). A reviewer of an earlier draft of this article pondered the need to educate ethics committee personnel on conceptualising intellectual disability and on current thinking around capacity.

Consent to participate in a research project ‘is only binding if it was given freely, voluntarily, and without undue influence [coercion]…[p]sychologists must ensure that the information is offered at a level which is in accordance with the client’s cognitive … abilities’ (Allan 2011:75). The world over, consent is valid if research participants have adequate information to make an informed decision; understand the information at a cognitive level; appreciate the situation and the consequences of the decision at an emotional level; have the ability to make a rational decision; make the decision freely and voluntarily; and can communicate their decision (Allan 2011; Carr, O’Reilly, Walsh, & McEvoy 2010). Meeting these requirements might pose significant challenges to the inclusion of intellectually impaired adults – even if such participation might be emancipatory and empowering (Barton, 2006; French & Swain 1997; London *et al*. 2011). But if we read this correctly, criteria relating to providing consent need not exclusively be measured by (a lack of) *capacity*, nor by verbal ability.

The current concept of consent is based on a dichotomous categorisation: people either have or do not have capacity to consent (Dye *et al*., 2004). A primary concern in the context of intellectual disability research is that participants may not understand what their involvement in a study entails, and are then unable to meet the criteria for providing informed consent (Allan 2011; Dye *et al*., 2004; London *et al*., 2011). Individuals with intellectual impairment may find it difficult to understand what research means, as well as the consequences of consenting or declining to participate. One reviewer of an earlier draft of this article brought to our attention, with helpful examples, the view that capacity to consent can vary according to the issue being addressed. For some research topics, a person could be deemed as having capacity to consent (e.g. regarding their views on where they live) whereas for other topics this is not possible (e.g. participating in the trial of an experimental drug).

In research involving child participants, good practice implies gaining children’s assent to participate in addition to the requirement of parental legal consent. To avoid the exclusion of potential participants with lower levels of comprehension and poorer ability to understand their involvement in research, and without referring to such persons as children, assent procedures can be initiated once consent is obtained from an authorised proxy or legal guardian.

Although obtaining consent from intellectually impaired participants should always be attempted first, the process can be conceptualised as being on a ‘sliding scale’ relative to the nature of impairment. Participants able to consent might be of ‘lesser’ intellectual impairment than participants able to assent (Ockert Coetzee, Personal communication at Alexandra Hospital, 2012 March 2). This can be illustrated as follows:

**Figure:**
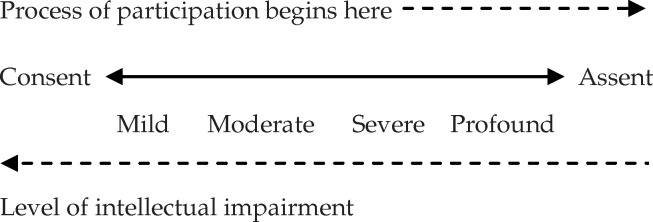


As far as the argument for *informed* consent goes, it needs to be recognised that ‘information alone is an inadequate predicate to meaningful choice’ (Grisso & Appelbaum 1998:14, in Cameron & Murphy 2006). Ill-explained options can be disabling – perhaps it is not so much the patient’s ability to consent that is most pertinent, but the researcher’s ability to explain options in a way that facilitates opportunities for making autonomous choices. It remains the researcher’s task to ensure that participants have been fully informed, that they know they have a choice to decline participation, are giving informed consent to participate, and understand the consequences of deciding on non-participation (Allan 2011; Inglis & Cook 2011).

Obtaining consent from intellectually impaired research participants should be a careful and lengthy process. Allan (2011) informs that, in order to communicate with participants about their involvement at a level that is non-discriminating and understandable without being derogatory, participants must have ‘enough time to make the decision’ and be afforded opportunities to ask questions and consult other people if they wish to (2011:75). In a similar vein to the ongoing monitoring of research ethicality post-approval by ethics committees as posited by Marzano (2007), Cameron and Murphy (2006) explain that consented participation is an ongoing process and not something established only at the beginning of contact. The greater participant control over consent at any point in the research process, the less likely it would be that research infringes the rights of participants with intellectual impairment (Cameron & Murphy 2006; Stone & Priestly 1996). But if one takes a position that it is unethical to exclude intellectually impaired individuals from participating in research, the formulation of solutions to difficulties regarding inclusion remains the researcher’s responsibility.

### Significance of work

Excluding intellectually impaired individuals from participating in research based on the argument of limited capacity can be unethical and a human rights violation. As coconstructors of studies that may affect them directly, adults living with intellectual impairment need not be excluded as coresearchers. By virtue of their expertise on the topic, their voice may stand in public and scientific service on disability matters.

## Conclusion

Excluding intellectually impaired individuals from participating in research based on the argument of limited capacity can be unethical and a human rights violation, especially in cases where effective measures have been put in place to assist eager individuals meet criteria for informed consent (Cameron & Murphy 2006; Gilbert 2004; Inglis & Cook 2011).

In upholding ethicality when considering intellectually impaired participants as coresearchers, there are a number of criteria to be mindful of. These include planning for a prolonged and continuous process of obtaining consent and assent; adapting information sheets and consent procedures appropriately whilst avoiding deprecating use of language; assessing each potential participant’s language skills in order to gauge individual levels of understanding; and communicating in participants’ home language(s), or having present a person familiar to the participant who can assist with translation and communication. Of further importance would be the ongoing conceptualisation and documentation of consent (and refusal); and establishing ways of initiating, maintaining, and terminating the research relationship. Care workers’ awareness of the research relationship also needs to be considered. Ultimately, the voluntary nature of participation and the participant’s right to make an autonomous decision about continuing or terminating involvement is paramount.

Apart from the core ethical principles of anonymity, confidentiality, and informed consent (Swartz *et al*. 2011), Kittay’s (2009) ‘fundamental ethical precepts’ of epistemic responsibility or empirical adequacy (know the subject participating in the research), and epistemic modesty (know, and admit to, what you don’t know) should also be considered. Refreshingly, researchers might do well to acknowledge and tolerate their own ignorance and lack of knowledge (see Walmsley 2004b). Every researcher ‘is still a person with [an own] stock of moral values and norms to be safeguarded’ and, for that matter, to be guarded against in harmony with established ethical principles (Marzano 2007:430). Ethical research practice should include peer and research supervision in addition to following relevant professional and statutory ethical codes (e.g. Ethical Rules of Conduct for Practitioners Registered under *The Health Professions Act* [*No. 56 of 1974*]). But despite containing codes, professional spaces, and guidelines, disability researchers will be required to embrace their ‘fear associated with the unknown and [be] willing to be vulnerable – not all-knowing [or] propped up by rules …’ (Swartz *et al*. 2011:4).

A disability research space will, as Marzano (2007) notes, always force researchers to come to terms with their own identity, to reflect on the nature of the social relations that they construct in the field, on the distribution of power within these, and especially on the legitimacy of their observations.

## Footnotes

1. No person, nor the state, may unfairly discriminate directly or indirectly against anyone on one or more grounds including … disability (RSA 1996).

2. Everyone has the right to freedom of expression, which includes freedom to receive or impart information or ideas and academic freedom and freedom of scientific research (RSA 1996).

3. Everyone has the right to have access to health care services (RSA 1996).

4. Everyone has the right to bodily and psychological integrity, which includes the right not to be subjected to medical or scientific experiments without their informed consent (RSA 1996).
